# Comparing adventitious root-formation and graft-unification abilities in clones of *Argania spinosa*

**DOI:** 10.3389/fpls.2022.1002703

**Published:** 2022-11-14

**Authors:** Pann Tzeela, Sela Yechezkel, Ori Serero, Avi Eliyahu, Sara Sherf, Yair Manni, Adi Doron-Faigenboim, Mira Carmelli-Weissberg, Felix Shaya, Vikas Dwivedi, Einat Sadot

**Affiliations:** ^1^ The Institute of Plant Sciences, Agricultural Research Organization-The Volcani Institute, Rishon LeZion, Israel; ^2^ The Robert H. Smith Institute of Plant Sciences and Genetics in Agriculture, The Robert H. Smith Faculty of Agriculture, Food and Environment, The Hebrew University of Jerusalem, Rehovot, Israel

**Keywords:** argan, adventitious roots, grafting, hormone profile, transcription profile

## Abstract

*Argania spinosa* trees have attracted attention in recent years due to their high resistance to extreme climate conditions. Initial domestication activities practiced in Morocco. Here we report on selection and vegetative propagation of *A. spinosa* trees grown in Israel. Trees yielding relatively high amounts of fruit were propagated by rooting of stem cuttings. High variability in rooting ability was found among the 30 clones selected. In-depth comparison of a difficult-to-root (ARS7) and easy-to-root (ARS1) clone revealed that the rooted cuttings of ARS7 have a lower survival rate than those of ARS1. In addition, histological analysis of the adventitious root primordia showed many abnormal fused primordia in ARS7. Hormone profiling revealed that while ARS1 accumulates more cytokinin, ARS7 accumulates more auxin, suggesting different auxin-to-cytokinin ratios underlying the different rooting capabilities. The hypothesized relationship between rooting and grafting abilities was addressed. Reciprocal grafting was performed with ARS1/ARS7 but no significant differences in the success of graft unification between the trees was detected. Accordingly, comparative RNA sequencing of the rooting and grafting zones showed more differentially expressed genes related to rooting than to grafting between the two trees. Clustering, KEGG and Venn analyses confirmed enrichment of genes related to auxin metabolism, transport and signaling, cytokinin metabolism and signaling, cell wall modification and cell division in both regions. In addition, the differential expression of some key genes in ARS1 vs. ARS7 rooting zones was revealed. Taken together, while both adventitious root-formation and graft-unification processes share response to wounding, cell reprogramming, cell division, cell differentiation and reconnection of the vasculature, there are similar, but also many different genes regulating the two processes. Therefore an individual genotype can have low rooting capacity but good graft-unification ability.

## Introduction

The argan tree (*Argania spinosa*) belongs to the Sapotaceae family; it is a wild tree, endemic to southwestern Morocco. The tree, which can live up to 200 years or more, is resistant to drought and high temperatures, grows in warm and arid areas characterized by non-fertile marginal soils, and has particularly deep roots; it is therefore considered capable of mitigating desertification processes (Khallouki et al., 2017). Argan is also known as the hardwood, or Moroccan iron tree; it withers and does not bear fruit during long periods of drought and regenerates during rainy periods (Morton and Voss, 1987). Argan trees have traditionally been used in the Berber culture to feed livestock and for oil production, mainly by female laborers (Lybbert et al., 2011; Khallouki et al., 2017). The oil produced from the seeds has great value in the fields of cosmetics and haute cuisine (Khallouki et al., 2017).

Argan trees were first brought to Israel in 1931 and planted in the botanical garden of the agricultural school Mikve Israel. Several attempts have been made to cultivate and grow argan since 1983, by germinating seeds of the three trees grown at Mikve Israel. Great variability in fruit yield was discovered among the trees in the first planted plots, reaching a maximum of 20–25 kg per tree after 6–7 years (Nerd et al., 1994). In addition, pollination self-incompatibility was found (Nerd et al., 1998), in agreement with other reports (Bellefontaine, 2010). A total of about 100 ha were planted with argan over the years; however, most of the plots were not successful economically, some were abandoned and today, only about 50 ha remain cultivated. The high genetic variability among the trees was reflected, among other things, in the high proportion of individual trees with poor yields, thus rendering the crop unprofitable. Surveys of the natural population of argan trees in Morocco indeed show that the species exhibits high heterozygosity (Louati et al., 2019), which is expressed in tremendous phenotypic differences between trees, including flowering phenology (Zahidi and Abdelhamid, 2015), yield, fruit shape, number and size of the kernels (Belcadi Haloui et al., 2017), and oil composition (Ait Hammou et al., 2019) and content (Ait Hammou et al., 2018). Failure to cultivate argan is due in part to the tree being very difficult to root (Nouaim et al., 2002; Justamante et al., 2017; Mazri et al., 2022); to date, no commercial cultivar varieties or proper rootstocks have been selected. Hence, the main obstacle in turning argan into a commercial crop on a significant scale is the difficulty in performing effective vegetative propagation of outstanding varieties.

In general, elite trees are propagated in a vegetative manner by rooting stem cuttings, either under tissue culture conditions or on rooting tables; alternatively, grafting is used. Both adventitious root regeneration after wounding (Druege et al., 2019; Lakehal and Bellini, 2019; Lakehal et al., 2019; Shi-Weng, 2021) and graft unification (Melnyk and Meyerowitz, 2015; Melnyk et al., 2015; Melnyk, 2017b; Melnyk, 2017a; Melnyk et al., 2018) involve the response to wounding, cell reprogramming, cell division, cell differentiation and vasculature fusion between either the root and the stem or the rootstock and the scion respectively. Adventitious roots are roots differentiated from non-root tissues. In trees, the founder cells can be of different origins such as the cambium, phloem parenchyma, inner cortical parenchyma, non-differentiated secondary phloem, cambium between the vascular bundles, or other cell types (Fahn, 1990). Following cell division, a root primordium is formed which grows through the upper cell layers and eventually emerges from the stem while its vasculature fuse with that of the stem (Riov et al., 2013). During grafting, first, secreted pectins are thought to lead to adherence between the scion and rootstock. Second, cell division leads to callus formation in the graft junction, which can originate from the cambium, phloem parenchyma and even the pith. Eventually the vasculature and cambium differentiate from the callus in accordance with existing tissues in the scion and rootstock (Melnyk, 2017b). Auxin plays an important role in both processes (Melnyk et al., 2015; Melnyk, 2017b; Lakehal and Bellini, 2019). Therefore, based on some specific indications in the literature, it is assumed that adventitious root formation and graft unification share some similar genetic pathways. For example, auxin response factor- ARF6 and ARF8 were found to play a role both in adventitious root formation (Gutierrez et al., 2009) and stem tissue reunion (Pitaksaringkarn et al., 2014). *Arabidopsis aberrant lateral root formation 4* (*alf4*) mutant plants barely make lateral roots (Sugimoto et al., 2010), and when grafted, exhibit phloem-reconnection malfunction (Melnyk et al., 2015). Tomato *Sl*WOX4 is expressed in phloem parenchyma cells that give rise to shoot-borne roots (Omary et al., 2022), and *Slwox4* mutant plants fail to form xylem fusions across a homograft (Thomas et al., 2022). In many plants, rooting ability declines during the juvenile-to-mature phase change, both in the sense of success rates and the length of time until success (Riov et al., 2013; Pizarro and Diaz-Sala, 2019), as does graft unification (Melnyk, 2017b). When adventitious roots were induced in difficult to root mature trees, some cell division occurred but not enough to form root primordia (Ballester et al., 1999; Greenwood et al., 2001; Vidal et al., 2003; Abu-Abied et al., 2012). Interestingly, failure to form enough callus to heal the graft junction is one of the most prominent signs of graft incompatibility in *Vitis vinifera* varieties grafted on hybrid rootstocks (Tedesco et al., 2022). Taking together, although adventitious root formation and graft unification are two different processes that culminate in different final organs, both processes rely on the regeneration capability of a single organ of a specific genotype, at a specific developmental stage.

Here we selected elite *A. spinosa* clones based on fruit yield from plots planted around the country. The trees’ ability to root was scored and found to be highly divergent. We took advantage of this collection to compare difficult- and easy-to-root clones for their ability to graft successfully.

## Materials and methods

### Materials

Chemicals such as Triton X-100, K-IBA, polyvinylpyrrolidone, dithiothreitol, standards of hormones, sodium acetate, formaldehyde, calcofluor white were from Sigma Aldrich. Solvents such as ethanol, methanol, isopropanol, acetic acid, were from Bio-Lab Itd. The suppliers of other specific chemicals are indicated below.

### Plant material, rooting and grafting

**Figure 1:**
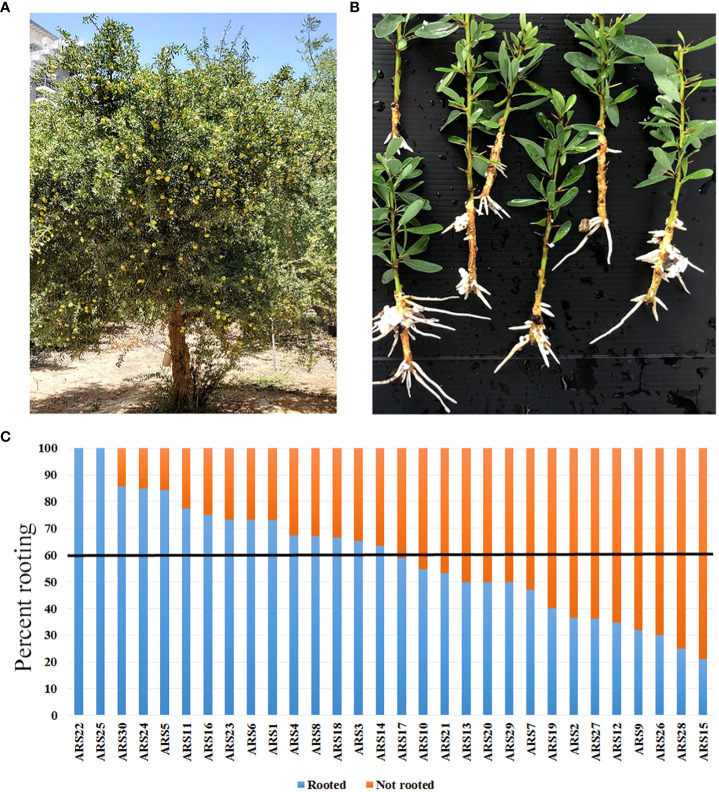
Distribution of rooting capacity among the 30 collected argan clones propagated by rooting of stem cuttings. **(A)** ARS1 tree. **(B)** Rooted cuttings from ARS1. **(C)** Percent rooting of all 30 clones. Black line marks 60% rooting: above this threshold, clones were considered easy to root.

Cuttings, about 4–5 mm thick and about 10 cm long, were harvested from branches of the current year’s growth from the trees in the field and later from the mother plants created and grown under optimal irrigation and fertilization conditions. Leaves and thorns were removed from the bottom one third of the cutting. The cuttings were treated with indole-3-butyric acid (K-IBA) at 6000 g/L for 1 min and incubated on a rooting table. Rooting medium consisted of 3:2:1 (v/v) styrofoam/vermiculite/peat. Tables were covered with nonwoven fabric and humidity was kept at 90% by fog sprayers operated for 10 s every 10 min from 6AM to 6PM. Rooting tables were heated to 25°C during the winter. The climate in the greenhouse was controlled by moist mattresses and fans. Rooting was scored after 2 and 3 months. The work presented in **Figure 1**; **Supplementary Figure 1** and **Supplementary Table 1** summarizes 5 years of tree collection and repetitive rooting experiments, for thousands of cuttings. For grafting; rootstocks were newly rooted cuttings after hardening (about 2-3 months post root induction). The scions were from the older mother plants, grown in the greenhouse as source of cuttings, and typically chosen to be a green branch of similar thickness as the rootstock. Briefly, the rootstock was incised in the middle to a depth of about 2 cm and the scion was cut to form a point. The scion, left with the top three leaf buds, was inserted into the groove in the rootstock and the graft area was wrapped in parafilm. The whole plant was then covered with a transparent plastic cover for 3–4 weeks until new leaves developed on the scion. Statistical analysis of the results was done using Chi square comparing two samples or One way Anova comparing multiple samples p<0.05 by the GraphPad Prism software.

### Histological analysis

The specimens were either the 1-1.5 cm of the cutting base or 1-1.5 cm of the grafting area. Fixation was performed in 7.4% formaldehyde, 50% ethanol, 5% acetic acid, 1% Triton X-100, v/v, 10 mg/mL polyvinylpyrrolidone (PVP), 1 mg/mL dithiothreitol (DTT) and 150 μg/mL ascorbic acid for 7 days at 4°C. The sample was then serially dehydrated in 50%, 70%, 90%, 100% and 100% absolute ethanol, each step for 24 h. Gradually increasing histoclear (K-clear – Kaltek t280)/ethanol concentrations were then applied (25%, 50%, 75%, 100%, 100%). Hista-Flex 8810/01 paraffin wax (Poth Hille & Co Ltd) was added gradually, first at room temperature and then after saturation at 42°C, and finally at 58°C with six repeat replacements of the paraffin. The samples were poured into molds and cooled. The blocks were dissected into 10- to 14-μm sections with a Leica RM2245 rotary microtome and dried on microscope slides. The paraffin was removed using 100% histoclear and samples were gradually rehydrated in 100%, 95%, 70%, 50% ethanol before staining in 1% safranin for 35 min. After a washing step in H_2_O, the samples were dehydrated again in 50%, 75%, 95% and 95% ethanol and stained with 0.3% Fast green for 3 min. After a short wash in 95% ethanol, the samples were dipped twice in clover oil for 3 min each time, and three times in histoclear for 3 min each time. The samples were then mounted under a coverslip using EUKITT mounting medium (Kaltek).

For cell wall staining, sections were cut manually with a razor blade and stained with 1% Calcofluor white (Sigma 18909).

### Microscopy

An upright Olympus BX53F microscope was used to image the histological sections, with UplanSApo 10 x 0.4 and 4 x 0.16 objectives. Calcofluor staining was imaged in a Leica SP8 confocal microscope with objective PL APO 10x/0.4, and a 2.2 mm working distance. Calcofluor was excited with a 405-nm laser and emission was captured between 430 and 460 nm.

### Preparation of hormones extract

Hormones were extracted at 0, 6, 24, 48, and 120 h after treatment of trees ARS1 and ARS7 with 6000 g/L K-IBA. Each treatment was performed in three biological repeats, and each repeat included eight cutting bases from which the bark was peeled as previously described (Ridoutt et al., 1995; Foucart et al., 2006). The bark samples including the cambium, phloem and cortical parenchyma were quickly frozen in liquid nitrogen and ground to a fine powder with mortar and pestle. The frozen powder (190–240 mg) was transferred to 2-mL Eppendorf tubes. Hormones were extracted in 1 mL of a cold 79% isopropanol, 20% methanol and 1% acetic acid (v/v) mixture containing 20 ng ^12^C-labeled internal standards as described below. The tubes were vortexed for 1 h at 4°C and then centrifuged at 14,000 rpm for 15 min. The supernatants were transferred to fresh 2-mL Eppendorf tubes. Two more extraction cycles were performed using 0.5 mL extraction solvent without the internal standards. The solvent was evaporated in a SpeedVac centrifuge and the pellets were dissolved in 200 µL of prechilled 50% methanol, centrifuged, and filtered through a 13 mm, 0.22-µm PVDF syringe filter into fresh 1.5-mL tubes. These ready-to-use extracts were kept at -20°C.

### LC–MS analysis

All LC–MS–MS analyses were conducted in a UPLC-triple quadrupole MS (Waters Xevo TQ MS). Separation was performed on a Waters Acquity UPLC BEH C18 1.7 µm 2.1 x100 mm column with a VanGuard precolumn (BEH C18 1.7 µm 2.1 x 5 mm).

Chromatographic and MS parameters were as follows: for ABA, auxin and cytokinin analyses, the mobile phase consisted of water (phase A) and acetonitrile (phase B), both containing 0.1% formic acid, in gradient elution mode. The solvent gradient program was as follows:

Time (min) Phase A (%) Phase B (%)

Initial 95 5

0.5 95 5

14 50 50

15 5 95

18 5 95

19 95 5

22 95 5

The flow rate was 0.3 mL/min and the column temperature was kept at 35°C. ABA, auxin and cytokinin analyses were performed using the ESI source in positive ion mode with the following settings: capillary voltage 3.1 kV, cone voltage 30 V, desolvation temperature 300°C, desolvation gas flow 565 L/h, source temperature 140°C. Quantitation was performed using MRM-Multiple Reaction Monitoring:

247/173, 247/187 for ABA, RT – 8.45, 253/206, 253/234 for d6-ABA, RT – 8.40

220/136, 220/202 for t-Z, RT – 2.44, 225/137, 225/207 for d5 t-Z, RT – 2.40

352/136, 352/220 for t-ZR, RT – 3.42, 357/137, 357/225 for d5 t-ZR, RT – 3.39

336/136, 336/204 for iPR, RT – 6.03, 342/137, 342/210 for d6 iPR, RT – 5.96

204/69, 204/136 for iP, RT – 4.88, 210/75, 210/137 for d6 iP, RT – 4.80

176/103, 176/130 for IAA, RT – 7.33, 181/106, 181/134 for d5 IAA, RT – 7.25

291/130, 291/134 for IAAsp, RT – 5.30, 297/134, 297/136 for DN IAAsp, RT – 5.25

305/130, 305/148 for IAGlu, RT – 5.73, 311/134, 311/150 for DN IAGlu, RT – 5.67

192/128, 192/146 for OxIAA, RT – 5.04

333/130, 333/186 for IBGlu, RT – 8.17

204/186, 204/144 for IBA, RT – 10.13, 208/190, 208/132, for d4 IBA, RT – 10.10.

LC–MS data were acquired with MassLynx V4.1 software (Waters). Quantification was performed using isotope-labeled internal standards (Sigma), except for OxIAA and IBGlu, which were quantified using calibration curves.

### RNA preparation

*A. spinosa* cutting base (1–1.5 cm) or graft zone (1.5-1  cm) was cut and peeled as described above. Three biological repeats with three plants each were used for the rooting or grafting zone at each time point (0, 24 h, 120 h after root induction or grafting), for a total of 30 repeats. The peel, containing the bark, cambium, phloem and cortical parenchyma, was frozen in liquid nitrogen. RNA (100 mg) was extracted from this tissue using the Norgen-Biotek RNA Extraction kit according to the manufacturer’s basic protocol, with several modifications. First, 100 mg was used. Second, a few grains of PVP-K30 were added to the lysis buffer in each tube, and third, after elution, RNA was precipitated in 3 M sodium acetate pH 5.5 and two volumes of cold ethanol at -20°C overnight. Before sequencing, RNA integrity number (RIN) analysis was performed by the Weizmann Institute of Science Biological Services and library preparation, as well as RNA sequencing, were performed at the Crown Genomics Institute of the Nancy and Stephen Grand Israel National Center for Personalized Medicine, Weizmann Institute, using a NovaSeq SP (200 cycles: 100 bp X2 paired-end data).

### Bioinformatics analysis

The raw-reads were subjected to a filtering and cleaning procedure. The FASTX Toolkit (Dobin et al., 2012)(http://hannonlab.cshl.edu/fastx_toolkit/index.html, version 0.0.13.2) was used to trim read-end nucleotides with quality scores <30, using the FASTQ Quality Trimmer, and to remove reads with less than 70% base pairs with a quality score ≤30 using the FASTQ Quality Filter. Clean-reads were aligned to the *Argania spinosa* reference genome extracted from the NCBI (GCA_003260245.1) using STAR software (v2.7.1a) (Trapnell et al., 2010) with an average mapping rate of 90%. Gene abundance was estimated using Cufflinks workflow (v. 2.2) (Trapnell et al., 2010). Principal component analysis (PCA) and heatmap visualization were performed using R Bioconductor (Gentleman et al., 2004), in which two exceptional repeats were omitted one of ARS1 24h R and one of ARS1 24h G. Differential expression analysis was done with the DESeq2 R package (Love et al., 2014). Genes that varied at least at one time point (0 vs. 24h, 0 vs. 120h, 24h vs. 120h at each plant group: ARS1_root, ARS7_root, ARS1_graft and ARS7_graft) more than twofold, with an adjusted *P*-value of no more than 0.05, were considered differentially expressed. Cluster analysis of the DEGs at each plant group at the 3 time points based on the average FPKM value, was conducted using the K-means algorithm using R function. Venn diagrams were calculated using “Venny” tool (Oliveros, 2007) or (http://bioinformatics.psb.ugent.be/webtools/Venn/ web tool) based on the Arabidopsis database (TAIR; https://www.arabidopsis.org/) homology accessions. The sequences of *Argania spinosa* proteins were used as a query term for searching the NCBI non-redundant (nr) protein database, which was carried out with the DIAMOND program (Buchfink et al., 2015). The search results were imported into Blast2GO version 4.0 (Conesa et al., 2005) for gene ontology (GO) assignments. Homologous sequences *via* blast tool (Altschul et al., 1990) were also identified by searching versus the *Arabidopsis* TAIR (https://www.arabidopsis.org/) database*, Malus domestica* (GCF_002114115.1_ASM211411v1_protein.faa) proteins and *Populus alba* (GCF_005239225.1_ASM523922v1_protein.faa) proteins. Gene Ontology (GO) and KEGG pathways enrichment analysis was performed using KOBAS 3.0 tool http://kobas.cbi.pku.edu.cn/kobas3/?t=1).

## Results

### Selection of clones and determination of their rooting ability

**Figure 2:**
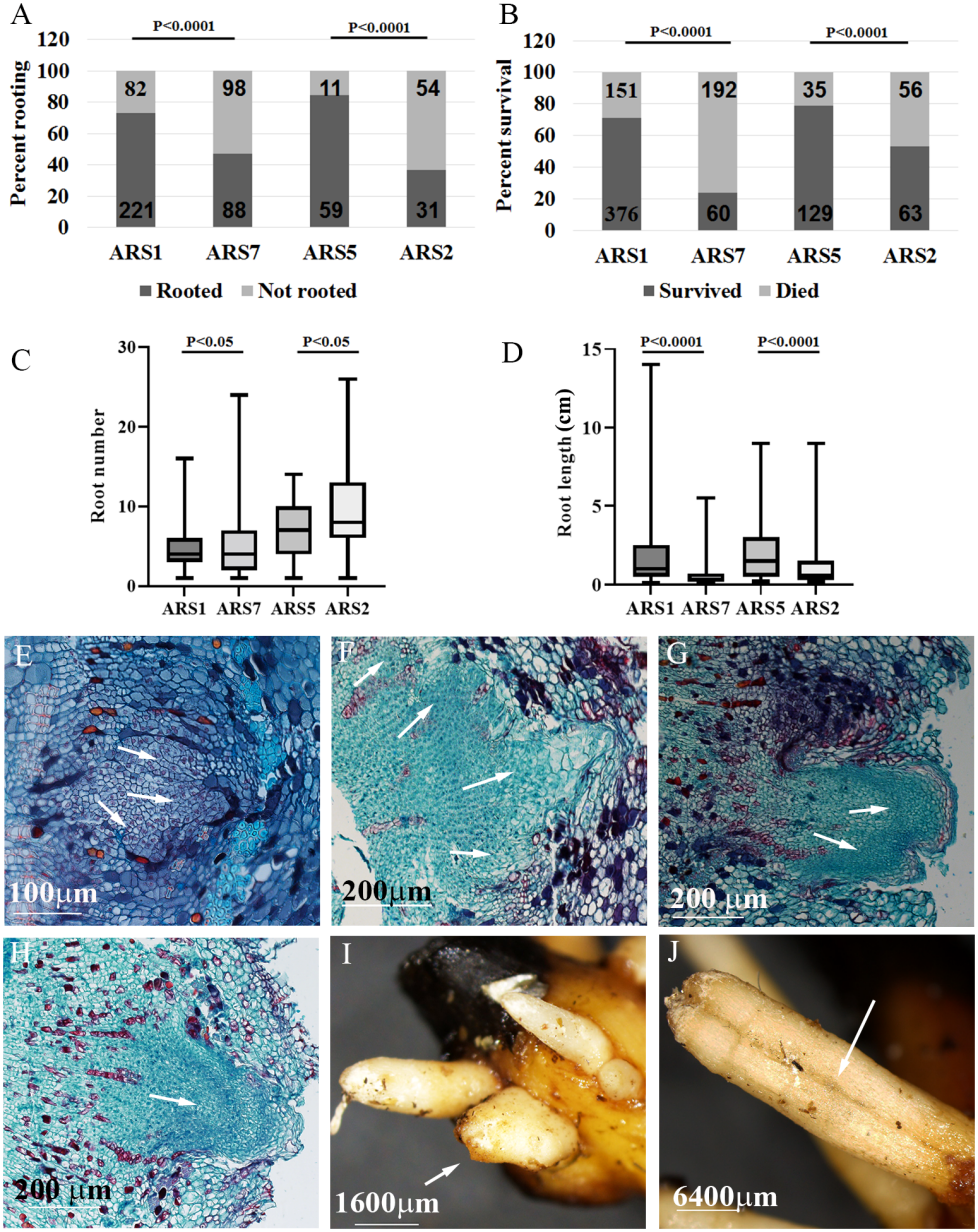
Specific comparison between difficult- and easy-to-root clones. Two pairs of trees, ARS7, ARS2 (difficult to root) and ARS1, ARS5 (easy to root), were compared. **(A)** Percent rooting. **(B)** Percent survival. Numbers in the bars are the number of cuttings. Statistical analysis: Chi square. **(C)** Average number of roots per rooted cuttings. **(D)** Average root length. Statistical analysis **(C, D)**: one-way ANOVA. Histological analysis of root primordia at different stages for ARS7 **(E–G)** and ARS1 **(H)**. Fused roots after emergence in ARS7 **(I)** and ARS2 **(J)**. Arrows in E-G show fused primordia, in H a single primordium and in I and J fused roots.

**Figure 3:**
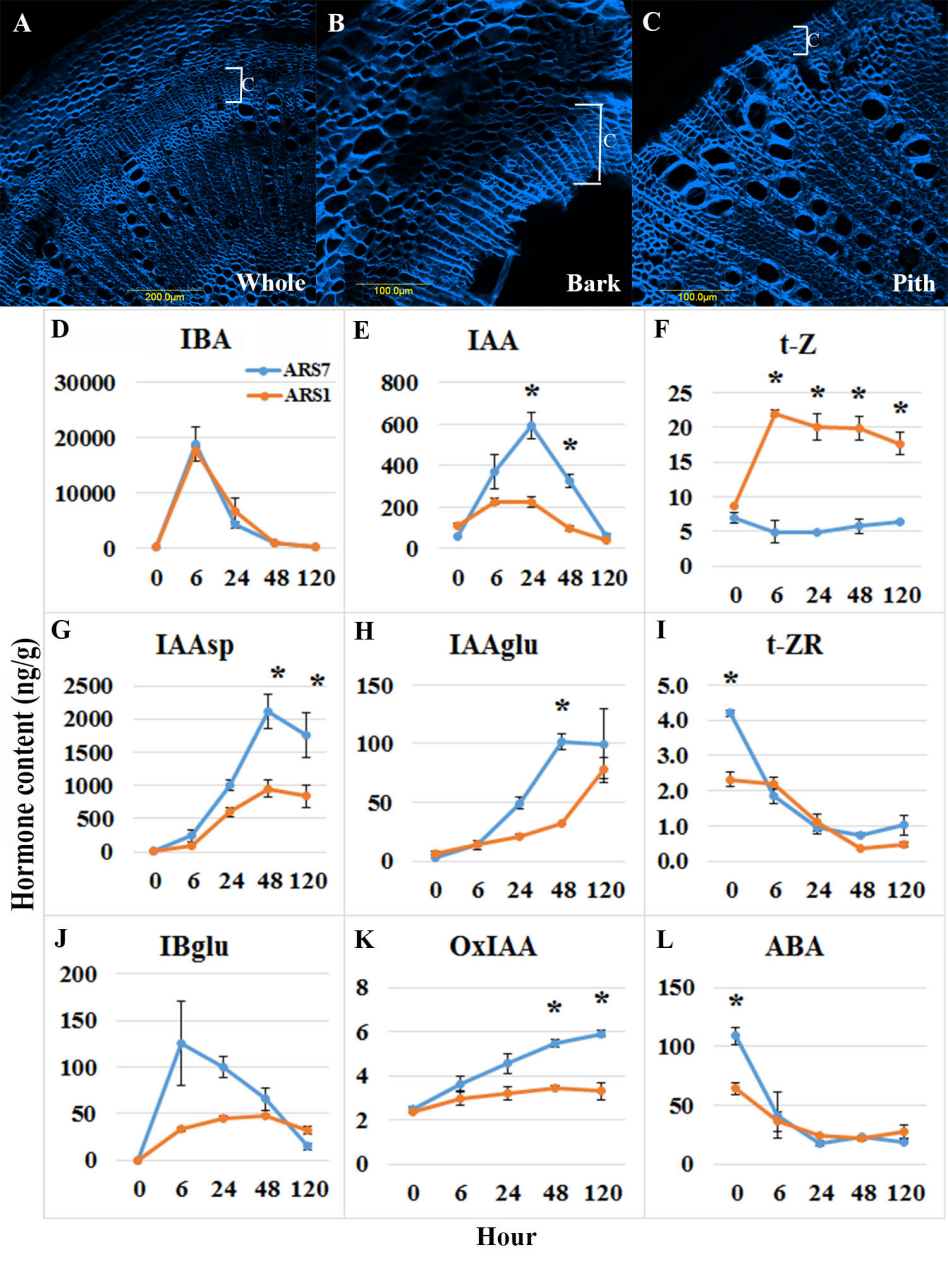
Hormone profiling. **(A–C)** Hand-cut sections and calcofluor staining showing enrichment of cambium, phloem and cortical parenchyma tissues. **(A)** Transverse section of the whole stem. **(B)** Bark after peeling, used for hormone profiling. **(C)** Residual inner part which was not used for profiling. C-cambium. **(D–L)** Hormone content analyses (ng/g wet weight) for the tissue in **(B)** from ARS1 and ARS7 trees. Asterisks show statistical significant difference between the samples at this time point *p*<0.05 One way Anova.

The high heterozygosity of *A. spinosa* necessitated a process of selection for varieties that are good for agriculture. Based on economic calculations taking into account expenses and income from the crop, it was suggested that an average annual yield of 30 kg per tree is the minimum for a commercial plantation. By weighing the fruit from each tree separately in several plots, we found that in an orchard of trees grown from seeds, only about 5–10% of all trees yielded this amount. Therefore, after searching the different argan orchards, 30 trees which were 7 to 10 years old and yielded at least 25–30 kg/year were selected and named varieties ARS1-ARS30 (**Figures 1A, B**, **Supplementary Figure 1** and **Supplementary Table 1**). The rooting ability of the clones ranged from 20 to 100% and was divided into two groups: difficult to root (<60%), and easy to root (≥60%) **Figure 1C**). Statistical analysis of selected samples showed significant differences between the rooting capabilities of clone ARS1 (easy) and ARS7 (difficult), and between clone ARS5 (easy) and ARS2 (difficult) (**Figure 2A**), and these were chosen for further investigation. Interestingly, survival rates of rooted cuttings were much lower for both difficult-to-root clones ARS7 and ARS2 (**Figure 2B**), suggesting a problem in the formed roots. Root counts showed more and shorter roots in ARS7 and ARS2 (**Figures 2C, D**). To get a better view of adventitious root formation in the different clones, histological analysis was performed. The bottom 1–1.5 cm of cuttings from clone ARS7 or ARS1 was fixed 30 days after pruning and treatment with auxin, when the cutting base started to swell. The tissue was embedded in paraffin and cut into thin sections. Notably, many of the root primordia of clone ARS7 which were found in the different sections had split tips or were fused (**Figures 2E–G**). In contrast, most of the adventitious root primordia in ARS1 looked normal (**Figure 2H**). Fused roots were also observed after root emergence (**Figures 2I, J**). Split or fused primordia/lateral roots have been previously described in *Arabidopsis* auxin transport and downstream auxin response mutants (Benkova et al., 2003; De Smet et al., 2010) and in poplar overexpressing WOX5 (Li et al., 2017). Therefore, we first measured auxin accumulation around the cambium—the tissue from which primordia emerge (**Figures 2E, G, H**). To enrich the sample for cambium cells, the bark was separated from the cutting base, which included most of the cambium tissue, the phloem and cortical parenchyma (**Figures 3A–C**). Hormones were extracted at 0, 6, 24, 48 and 120 hrs after IBA treatment and analyzed by LC–MS–MS (**Figures 3D–L**). IBA was found to accumulate in a similar manner in both trees, with a peak at 6 h and a gradual decline to 120 h (**Figure 3D**). Interestingly, while ARS7 accumulated more IAA with a peak at 24 h (**Figure 3E**), ARS1 accumulated more cytokinin in the form of trans-zeatin (t-Z), exhibiting constant high levels from 6 h until 120 h (**Figure 3F**). The inactive form (t-ZR) was initially higher in ARS1 but became equal in both trees after 6 h (**Figure 3I**). Following the higher accumulation of IAA in ARS7 at 24 h, its conjugates peaked at 48 h (**Figures 3G, H**) and OxIAA continued to accumulate, even at 120 h, in this tree (**Figure 3K**). IBglu accumulation (in ARS7) overlapped with that of IBA after 6 h (**Figure 3J**). ABA was initially higher in ARS7 but after treatment became equal in both trees (**Figure 3L**). Taken together, the significant difference in the auxin-to-cytokinin ratio between the two trees likely contribute to the difference in their rooting capabilities.

### Reciprocal grafting of easy- and difficult-to-root clones

**Figure 4:**
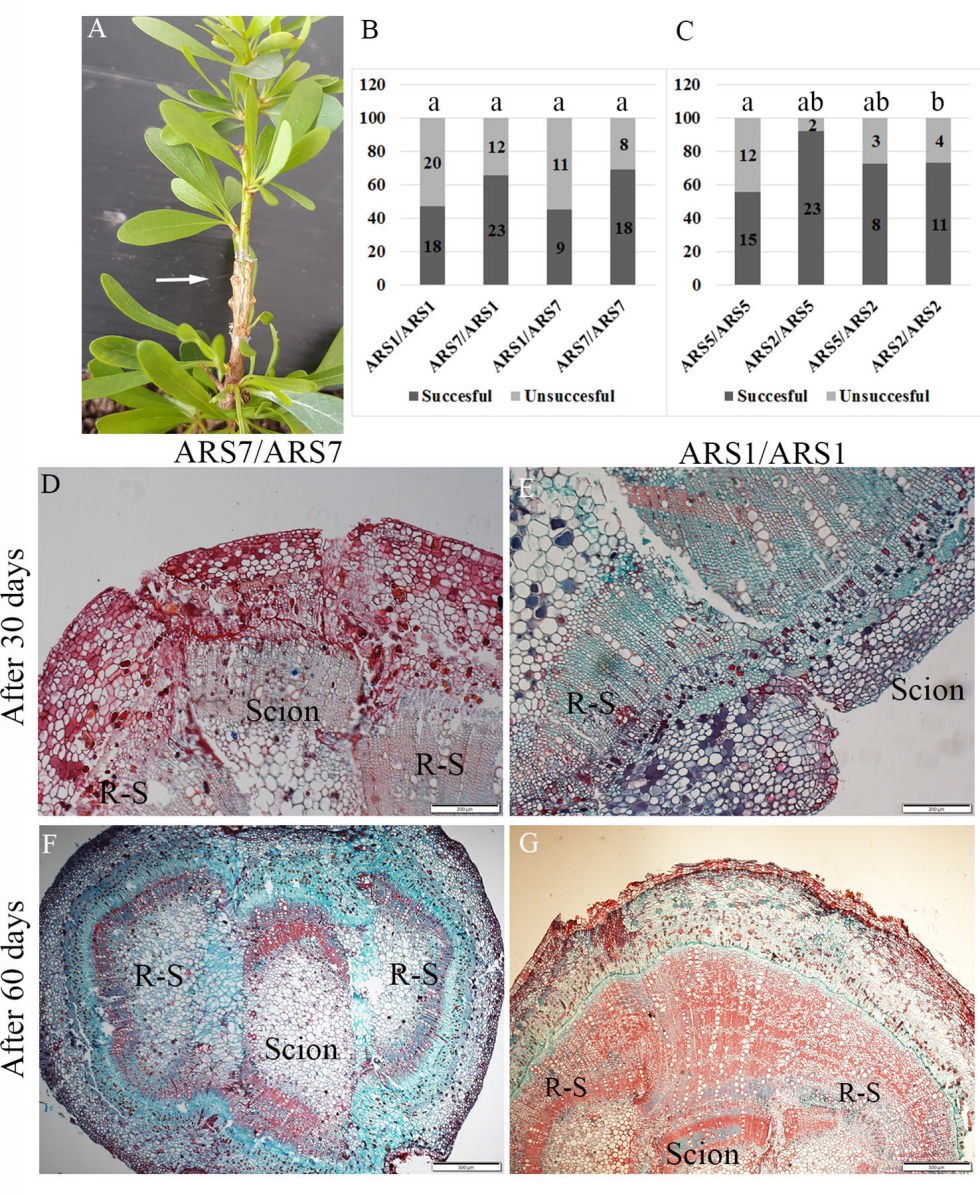
Reciprocal grafting of ARS1 and ARS7 or ARS5 and ARS2. **(A)** Successful grafting of ARS7/ARS1. **(B, C)** Results of all scion/rootstock grafting combinations. Numbers in the bars are number of repeats. Statistical analysis: Chi square, *p* < 0.05. **(D–G)** Histological analysis of the graft-unification zone in self-grafted ARS7/ARS7 and ARS1/ARS1 after 30 days **(D, E)** and 60 days **(F, G)**. R-S=rootstock.

Many fruit trees are grown on rootstocks which provide important traits, such as resistance to harsh soils and diseases, increased yield, dwarfism and more (Goldschmidt, 2014). Grafting is also a common solution for the propagation of difficult-to-root plants (Hartmann et al., 2011). Grafting success depends on the taxonomic relationships between the scion and rootstock, the closer is the better; with high rate of success in inter species, less in inter genus, and even less in inter family combinations (Goldschmidt, 2014). However, in some cases graft incompatibility was found among varieties of the same species (Chen et al., 2017). We tested whether ARS1 and ARS5 can serve as rootstocks for ARS7 and ARS2 respectively, and addressed the question of whether difficulty in rooting is a sign of low regeneration ability which is also reflected in a low rate of graft success. Self and reciprocal grafting were performed between the two pairs, ARS1 and ARS7, or ARS5 and ARS2, i.e., all served as either the scion or the rootstock or both. Success of grafting was scored according to emergence of new leaves from the scion after 30–45 days (**Figure 4A**). Rate of grafting success was 45–69% for all scion/rootstock combinations of ARS1/ARS7 and 56–92% for all combinations of ARS5/ARS2. No advantage was observed for self-grafting of the easy-to-root clones—ARS1/ARS1 or ARS5/ARS5—compared to the difficult-to-root clones—ARS7/ARS7 and ARS2/ARS2 (**Figures 4B, C**). It is known that partial graft unification can lead to the appearance of incompatibility phenotypes after a few years (Loupit and Cookson, 2020). To further check the graft-unification process, histological analysis was performed. Transverse sections were collected from the graft zone of ARS1/ARS1 and ARS7/ARS7 after 1 and 2 months. **Figures 4D-G** shows that whereas after 1 month, callus layers were observed at the connection planes between the rootstock and the scion, after 2 months, good unification occurred, forming a connection between the different tissues. Taken together, our data suggest that the mechanism that causes the rooting difficulty does not impair grafting capacity in ARS7.

### Comparative transcriptome profiling during rooting and grafting

**Figure 5:**
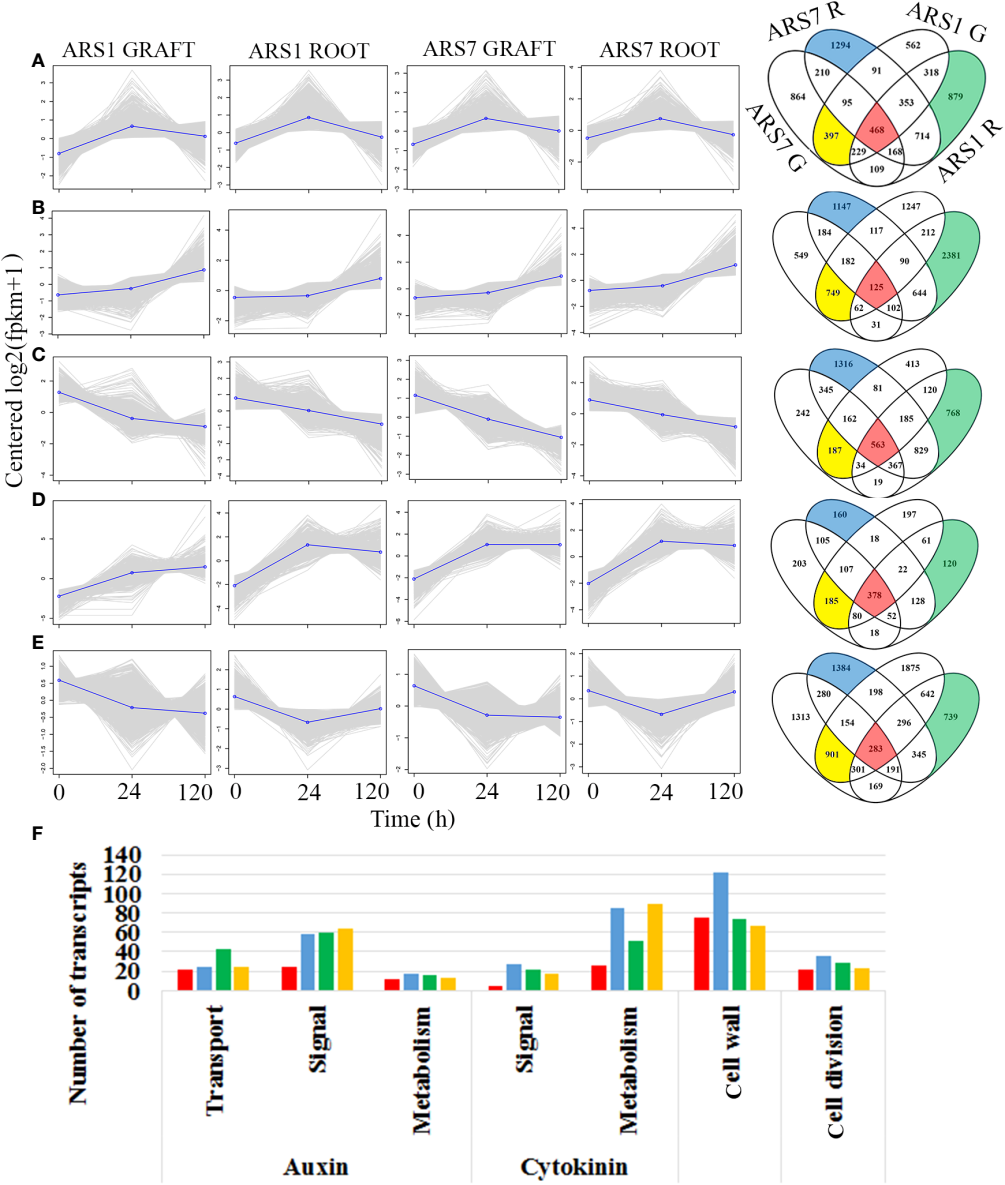
Bioinformatics analysis of the RNA-seq results. **(A–E)** RNA was prepared in three biological repeats each composed of three plants. R, rooting tissue; G, grafted tissue. The transcripts in the different groups were clustered and the content of similar or different transcripts was analyzed by Venny software in the groups showing similar cluster patterns. Red, transcripts common to rooting and grafting zones in the two trees; yellow, transcripts specific for grafting in both trees; blue and green, transcripts specific for the rooting zone in ARS7 and ARS1, respectively. **(F)** KEGG analysis of gene ontology according to colored zones in the Venn diagrams.

**Figure 6:**
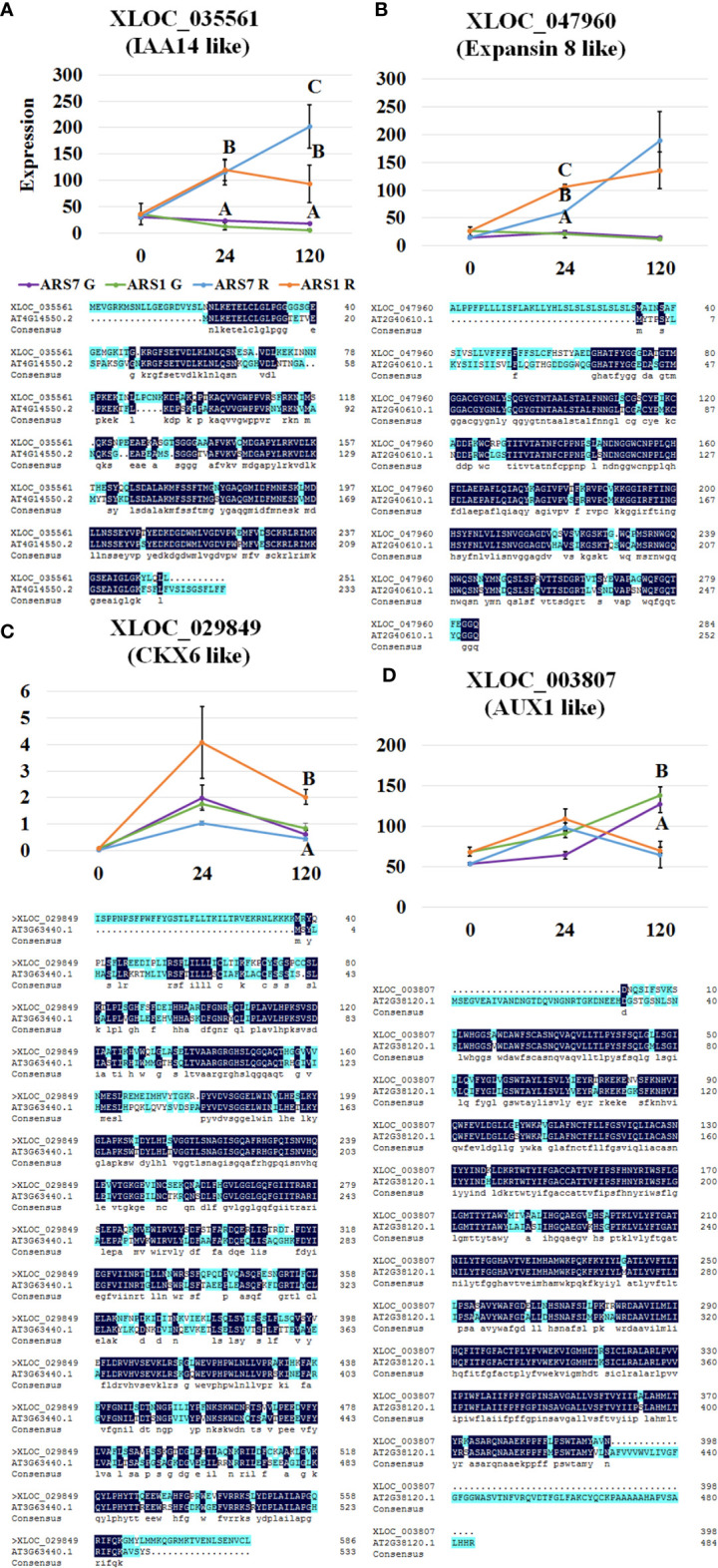
Expression profiles of specific genes. Expression levels were determined as number of reads in the RNA-seq using three repeats for each treatment. The best assembled hit for each transcript was translated and the amino acid sequence was aligned to that of the corresponding *Arabidopsis* transcript. G, grafted tissue; R, rooting tissue. **(A)** Expression of solitary root IAA14-like. **(B)** Cytokinin oxidase/dehydrogenase-like (CKX6-like), which participates in cytokinin catabolism. **(C)** Expansin 8-like, involved in cell wall loosening. **(D)** Auxin influx transporter AUX1-like. Different letters show statistically significant difference by one-way ANOVA at *p* < 0.05.

To further explore the differences and similarities in the mechanisms underlying rooting and grafting in ARS1 and ARS7, RNA deep sequencing was performed. RNA was extracted in three biological repeats from tissues enriched in cambium–phloem–cortical parenchyma (**Figures 3A–C**), either from the self-graft zone (labeled G) or the cutting bases after treatment with IBA (labeled R) of ARS1 and ARS7, at time 0, 24 h and 120 h post-treatment. These time points were chosen to cover short- and long-term responses, as previously described (Notaguchi et al., 2020; Ranjan et al., 2022). The average number of reads was more than 19 million/sample, mapped at 90% on average (**Supplementary Table 2**) to the recently published argan genome (Khayi et al., 2020). Principal component analysis (PCA) showed that expression was mainly influenced by the clone (**Supplementary Figure 2A**) or the time when samples were harvested (**Supplementary Figure 2B**). More differentially expressed genes (DEGs) at each time point between the ARS1 and ARS7 clones (≥2-fold, *p*_adj_ < 0.05) were found related to rooting (9669) than to grafting (7800) (**Supplementary Table 3**). When the transcripts were sorted into those with significantly changed expression (≥2-fold, *p*_adj_ < 0.05) between the different time points, there were more common transcripts related to grafting than to rooting between ARS7 and ARS1 (**Supplementary Figure 3**). Taken together, the expression profile suggested a larger difference between ARS1 and ARS7 related to rooting than to grafting, which was in agreement with our other findings (**Figures 2**, **4**). Next we looked for transcripts that are equally expressed in the rooting or grafting domains at the tested time points using cluster analysis. Transcripts exhibiting a change in expression in response to the treatment (≥2-fold *p*adj < 0.05) were divided into four groups: ARS1 R, ARS1 G, ARS7 R, and ARS7 G. Each group of transcripts underwent clustering according to expression patterns. Clusters with a similar pattern of expression from the four groups were compared for transcript content by Venn analysis (**Figures 5A–E**). A total 1817 transcripts had a similar pattern of expression in the fractions enriched with cambium–phloem–cortical parenchyma from the two trees in both the graft and rooting domains (**Figure 5**, red zones in the Venn diagram and **Supplementary Table 4**), consisting of 6% of the total number of DEGs. A total 5301 transcripts were sorted as specific to ARS7 rooting (**Figure 5**, blue zone in the Venn diagrams and **Supplementary Table 5**), and 4996 for ARS1 rooting (**Figure 5**, green zone in the Venn diagrams and **Supplementary Table 6**). The yellow domains contained 2419 transcripts that were significantly changed only during grafting in both trees (**Supplementary Table 7**). KEGG analysis showed enrichment of DEGs related to auxin metabolism, transport and signaling, cytokinin metabolism and signaling, cell wall modification and cell division (**Figure 5F**). Among the rooting-specific transcripts (blue and green regions in **Figure 5**), we looked for those that appeared in two different cluster groups, necessarily showing different patterns of expression in the two trees during rooting. A total 884 common transcripts were found by the Venn analysis between the green and blue zones. **Figure 6** shows some representative transcripts as examples, the sequences of which were compared at the amino acid level to that of the corresponding gene from *Arabidopsis* to verify the annotation. **Figures 6A–C** shows the expression profile of IAA14-like, CKX6-like and expansin 8-like, representing the auxin signaling, cytokinin metabolism and cell wall-modifying protein categories, respectively. The three showed equal expression in the graft zone but significantly different expression profiles in the rooting zone of the two trees. The auxin influx transporter AUX1-like exhibited different expression patterns in the rooting and grafting zones, but equally between the trees (**Figure 6D**). Taken together, our data suggest that in argan, while there are similar signaling pathways underlying rooting and graft unification which are regulated by similar genes with similar expression profiles, these form a small fraction (6%) of the total number of genes involved. A larger fraction of transcripts regulating rooting or grafting correspond to different genes, or similar genes with different expression patterns.

## Discussion

Unlike many edible fruit and nut trees, *A. spinosa* has not yet experienced intensive domestication and cultivation activity, including breeding, selection and vegetative propagation, thereby reducing genetic variability (Miller and Gross, 2011). As a result, this species has retained its naturally high heterozygosity (Louati et al., 2019). Thus, the collection of trees described here provides an interesting and unique biological system to study differences in adventitious root regeneration capability in different clones of the same species that are still so different from each other. While the trees were selected according to their high fruit yield, their rooting capability was highly divergent. The significant difference in rooting capability between ARS1 and ARS7 was accompanied by a difference in the accumulation of auxin and cytokinin shortly after root induction. Whereas the difficult-to-root clone ARS7 accumulated more IAA, the easy-to-root clone ARS1 accumulated more cytokinin at the cutting base, specifically in the cambium–phloem–cortical parenchyma-enriched fraction. While root primordia start to appear much later in the trees, these short-term changes likely regulate cell reprogramming and the determination of adventitious root founder cells (Druege et al., 2019; Lakehal and Bellini, 2019; Díaz-Sala, 2020). Auxin is the major plant hormone promoting adventitious root formation and in some cases, its accumulation is associated with better rooting capability. For example, in *Eucalyptus grandis*, higher levels of IAA were found in juvenile easy-to-root cuttings than in mature difficult-to-root ones (Abu-Abied et al., 2012). A similar observation was made in *Pisum sativum* (Rasmussen et al., 2015). However, other species, such as chestnut (Ballester et al., 1999; Vidal et al., 2003), hazelnut (*Corylus americana* × *C. avellana*) and elm (*Ulmus americana*) (Kreiser et al., 2016), show no specific correlation between high endogenous IAA levels and better adventitious root formation. Of note, easy-to-root elm cultivars were more efficient in converting IBA to IAA, as determined by using isotope-labeled IBA (Kreiser et al., 2016). At this point, the source of the higher IAA accumulation in ARS7 is not clear, although higher conversion of IBA to IAA (Zolman et al., 2000) or a higher rate of biosynthesis (Casanova-Sáez et al., 2021) might be assumed. A fine-tuned balance between auxin and cytokinin is known to be important for post-embryonic organogenesis (Ikeyama et al., 2010) and to be influenced by wounding, which itself can promote biosynthesis of both auxin and cytokinin (Ikeuchi et al., 2019). In addition, it has been shown that establishment and maintenance of the vascular cambium itself, where adventitious roots are formed, involves the coordination of auxin and cytokinin and their complex effects on each other’s signaling pathways (Wang et al., 2021). On the other hand, accumulated data suggest that cytokinin inhibits adventitious root formation (Lakehal and Bellini, 2019), and it was therefore surprising to find that the easy-to-root clone accumulated more cytokinin than the difficult-to-root one, the latter accumulating more auxin. The striking difference in IAA-to-cytokinin ratio between the two trees might result from the response to wounding, response to the IBA treatment, or the mutual interaction between them, and it might be specific to the time window of the analysis. This difference might have contributed to the high frequency of abnormal root primordia in ARS7 observed in the histological analysis, and the low rate of survival of rooted cuttings compared to ARS1. It also suggests that different molecular mechanisms underlie rooting recalcitrance in plants. For example, in *Eucalyptus globulus*, an easy-to-root clone accumulated IAA, IAAsp and cytokinin, whereas a difficult-to-root clone accumulated IAA to similar levels, but significantly less cytokinin and IAAsp (Negishi et al., 2014). In pea, it was found that while juvenile easy-to-root cuttings accumulate IAA in the short term (6–24 h) and cytokinin in the long term (96 h), the mature difficult-to-root cuttings accumulate significantly less IAA in the short term and less cytokinin in the long term (Rasmussen et al., 2015). *Picea abies* hypocotyls that rooted better after exposure to red light accumulated less IAA and less cytokinin than hypocotyls exposed to white light (Alallaq et al., 2020). Therefore, other difficult-to-root clones of *A. spinosa* might exhibit yet a different pattern of IAA-to-cytokinin ratios during adventitious root induction compared to ARS7.

The big difference in rooting capability expressed in the cutting bases of ARS1 and ARS7 was not reflected in the upper side of similar branches when they were grafted and showed a similar rate of successful graft unification. Although the two processes share similar scenarios—wounding, cell reprogramming, cell division, and cell differentiation toward reconnection of the vasculature, they have evolved different complex biologies. Indeed it was shown that when the inflorescence stem of *Arabidopsis* is cut, differences exist in gene expression between the parts above and below the cut (Asahina et al., 2011), i.e., in tissue that was once identical. In the case of rooting and grafting, other differences include treatment with auxin only to the cutting base and tissue tightening only to the upper part of the stem. In addition, while rooting involves *de-novo* differentiation of a whole organ, graft unification involves reconnection of existing tissues. Of note, a difficult-to-root clone of *Eucalyptus globulus* was also impaired in its response to gravitational signals (Negishi et al., 2014), suggesting a general problem in the polar transport of auxin affecting both processes. To shed more light on the differences and similarities in the molecular mechanisms underlying rooting vs. grafting in the two clones of *A. spinosa* studied here, ARS1 and ARS7, we performed comparative RNA-seq analysis during root induction and self-grafting. A comparison of DEGs between the two trees showed that the group of DEGs related to rooting was larger than that related to grafting. This correlated with the finding that the trees differ in their rooting capability but not in their graft-unification ability. At the same time, 6% of the DEGs changed in a similar manner in the two trees in both the rooting and grafting zones (marked in red in **Figure 5**). Among these were transcripts related to auxin, cytokinin, cell wall modification and cell division. This result is in agreement with the assumption that there may be a relationship between rooting ability and graft-unification ability. Of note, the KEGG analysis revealed a main difference in auxin transport and cell wall related transcripts between ARS1 and ARS7 rooting zones. To gain more information on the transcripts potentially underlying the difference in rooting capability between ARS1 and ARS7, we focused on some specific ones. IAA14 -like, for example, was significantly upregulated in ARS7 compared to ARS1 after 120 h. In *Arabidopsis*, an IAA14 gain-of-function mutation, termed solitary root, has a role in suppressing cell-cycle progression during lateral root formation; thus, plants carrying this mutation have a primary root with no lateral roots (Fukaki et al., 2002) and adventitious root formation from their hypocotyls is inhibited (Li et al., 2021). Following the peak of auxin at 24 h (**Figure 3**), it was expected that IAA14-like protein would undergo ubiquitination and degradation (Dharmasiri and Estelle, 2004). However, here, at the transcriptional level, there was a significant difference between the two argan clones, with the difficult-to-root one expressing higher levels of IAA14-like, which potentially contributed to the inhibition of adventitious root formation. Of note, the proline which is replaced to serine in solitary root, *slr1*, rendering it more stable (Fukaki et al., 2002) is conserved in both trees (**Supplementary Figure 4**). The difference in IAA14-like expression occurred on a background of differences in auxin-to-cytokinin ratio which were also expressed in the differential expression of related transcripts, for example cytokinin oxidase/dehydrogenase (CKX6)-like which potentially leads to cytokinin breakdown (Werner et al., 2003). CKX6-like showed significantly higher expression in ARS1, which also accumulated more cytokinin, suggesting a difference in the machinery regulating cytokinin metabolism. The differential auxin/cytokinin regulation was accompanied by, or contributed to the regulation of the differential context of cell wall modification-related transcripts. This was exemplified here by the expression of an expansin-like transcript showing significantly higher expression after 24 h in ARS1 compared to ARS7. The mechanical property of cell walls is important for lateral organ development in plants (Landrein and Hamant, 2013; Vilches Barro et al., 2019; Duman et al., 2020; Pizarro and Díaz-Sala, 2021) and specifically, expansin has been found to play a role in lateral root formation (Ramakrishna et al., 2019). Of note, the above three transcripts were significantly changed between the trees only at the rooting site but not at the grafting site. To exemplify the expression profile of another important transcript, we showed that expression of the auxin-influx transporter AUX1-like decreases in the rooting zone but further increases in the grafting zone after 120 h in a similar manner in both trees.

Taken together our data suggest that rooting capability is the end result of multiple coordinated changes involving, among other things, the ratio of auxin to cytokinin accumulation and downstream signaling with delicate fine-tuned changes in cell wall properties. A different balance of the same parameters is required for graft unification, which involves some overlapping as well as different transcripts. In this respect, it is interesting to note that rejuvenation which restores rooting ability can be achieved by sequential grafting (Huang et al., 1992), suggesting that branches that have lost rooting capability can still function as scions and gain the missing, albeit unknown traits to resume rooting ability from the rootstock. To further decipher the exact signaling pathways common to rooting and grafting, it will be necessary to use model plants that can be easily genetically modified.

## Data availability statement

The data presented in the study are deposited in NCBI repository, accession number PRJNA863910.

## Author contributions

PT conducted all experiments, SY, OS, AE, and SS rooting, YM planting trees, AD-F bioinformatics, MC-W and FS hormone analysis, VD lab work, ES planned the experiments and wrote the paper. All authors contributed to the article and approved the submitted version.

## Funding

We thank the chief scientist of the ministry of Agriculture for funding grant number 20-01-0270.

## Acknowledgments

We thank the following people for the argan elite clones found in their orchards: Prof Y. Mizrahi for ARS1, Z. Gilad. P. Snir and A. Stromsa for ARS2-ARS8, Dr. E. Solway and N. Solway for ARS9-ARS15, J. Nessim for ARS16-ARS21, A. Landau for ARS22-ARS26, and E. Sade for ARS27-ARS30. We thank the following people for planting experimental plots using trees propagated during this work: Dr. G. Ben-Ari, Y. Kosto, R. Rotcshild, M. Levi, Y. Karon and J. Nessim. 

## Conflict of interest

The authors declare that the research was conducted in the absence of any commercial or financial relationships that could be construed as a potential conflict of interest.

## Publisher’s note

All claims expressed in this article are solely those of the authors and do not necessarily represent those of their affiliated organizations, or those of the publisher, the editors and the reviewers. Any product that may be evaluated in this article, or claim that may be made by its manufacturer, is not guaranteed or endorsed by the publisher.

## Supplementary material

Supplementary Figure 1 Photos of all 30 selected and propagated trees.

Supplementary Figure 2 PCA of the RNA-seq results.

Supplementary Figure 3 Venny analysis of the number of common and different DEGs with time, comparing the rooting zone to grafting zone in the ARS1 and ARS7 trees.

Supplementary Figure 4 Sequence analysis around the *slr1* P/S mutation in both trees.

Supplementary Table 1 List of trees, original names, and places and year of collection.

Supplementary Table 2 Number of reads and percent mapping to the argan genome for all 30 samples.

Supplementary Table 3 Number of DEGs between the two trees related to the two different zones of rooting and grafting.

Supplementary Table 4 List of transcripts in the red zones in **Figure 5**.

Supplementary Table 5 List of transcripts in the blue zones in **Figure 5**.

Supplementary Table 6 List of transcripts in the green zones in **Figure 5**.

Supplementary Table 7 List of transcripts in the yellow zones in **Figure 5**.
