# Supplementary figures and images for: Comparing adventitious root-formation and graft-unification abilities in clones of Argania spinosa
Source: Front Plant Sci. 2022 Nov 14;13:1002703. doi: 10.3389/fpls.2022.1002703 (PMC9702570; doi:10.3389/fpls.2022.1002703)

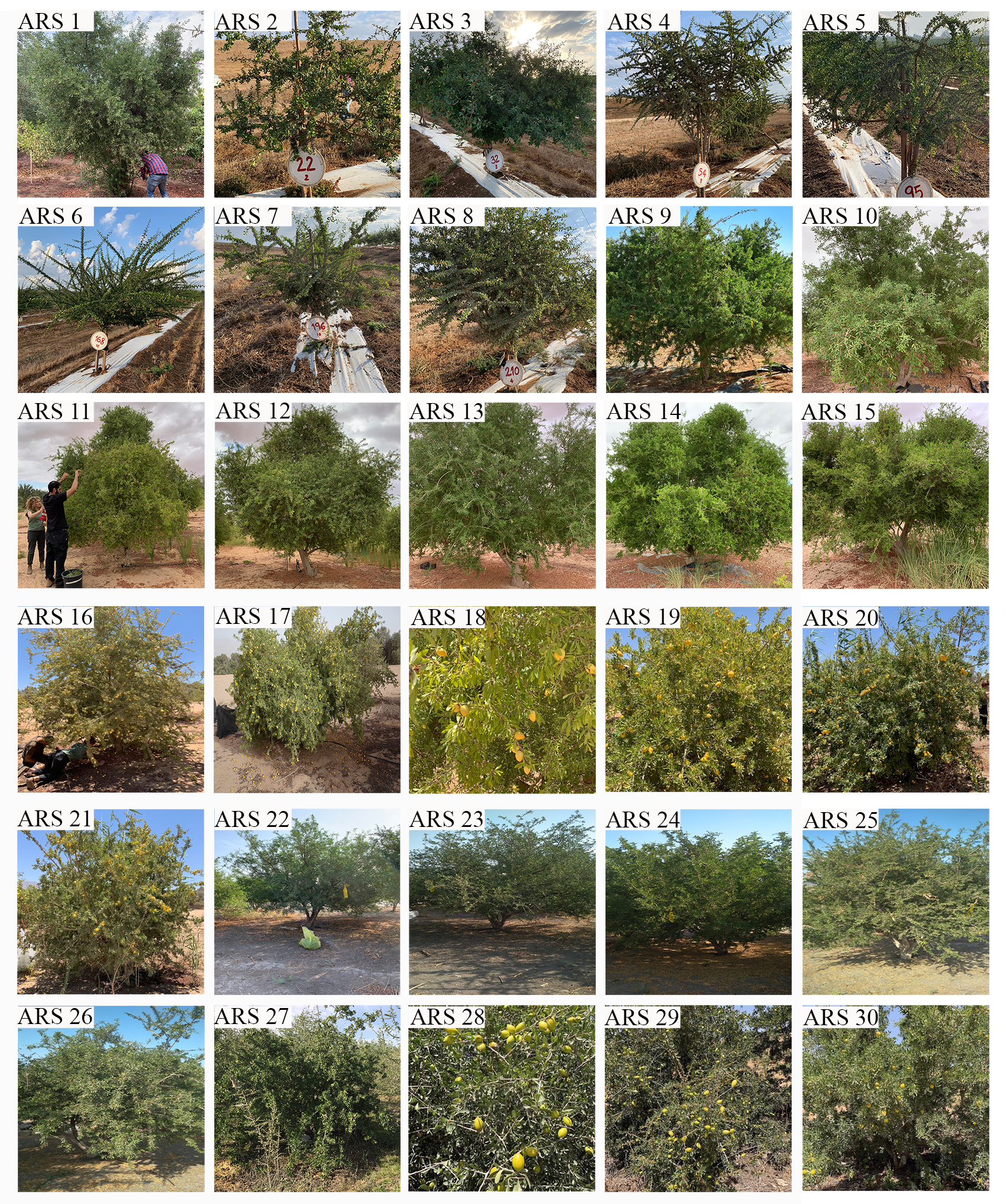

Supplement: Supplementary Figure 1 — Photos of all 30 selected and propagated trees. [file Image_1.tif]

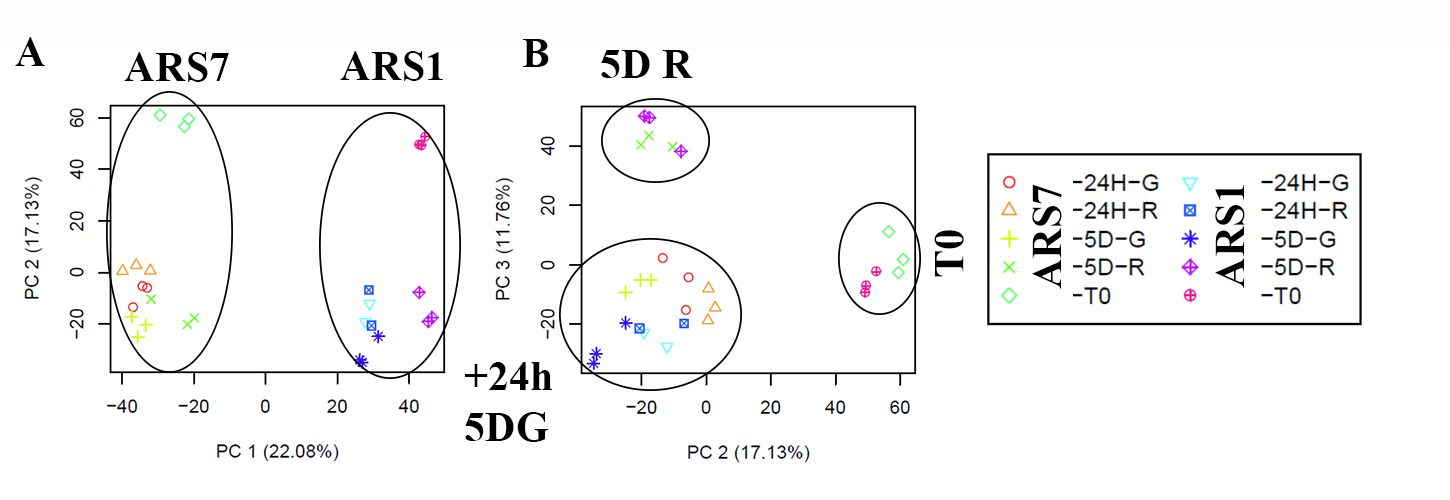

Supplement: Supplementary Figure 2 — PCA of the RNA-seq results. [file Image_2.tif]

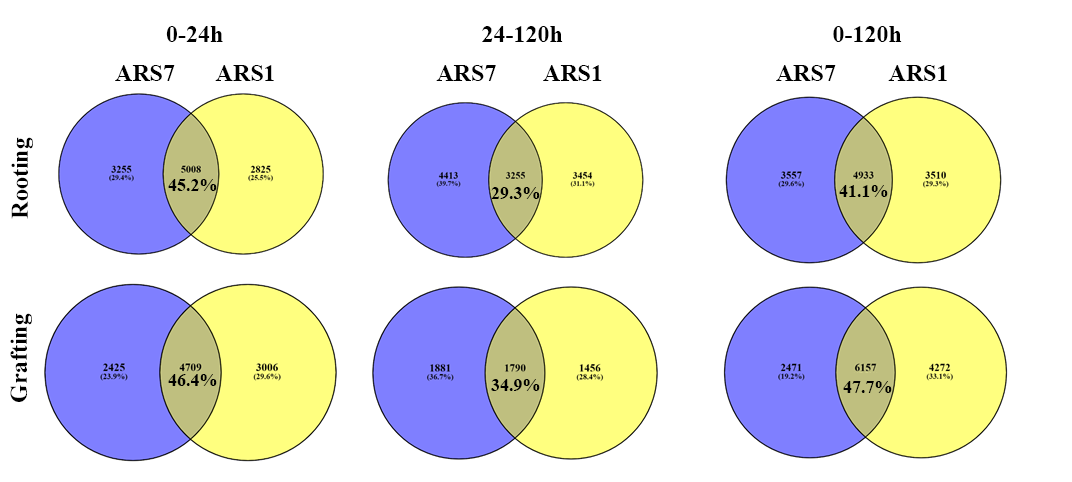

Supplement: Supplementary Figure 3 — Venny analysis of the number of common and different DEGs with time, comparing the rooting zone to grafting zone in the ARS1 and ARS7 trees. [file Image_3.tif]

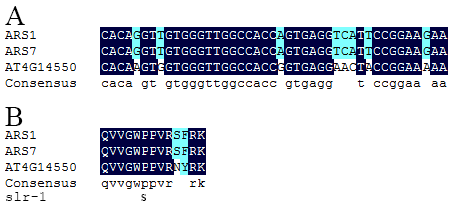

Supplement: Supplementary Figure 4 — Sequence analysis around the slr1 P/S mutation in both trees. [file Image_4.tif]
